# The association of Ned Kelly tattoos with suicide and homicide in a forensic context—a confirmatory prospective study

**DOI:** 10.1007/s12024-023-00655-w

**Published:** 2023-05-23

**Authors:** Roger W. Byard, Hamish Maxwell-Stewart

**Affiliations:** 1 Forensic Science SA , The University of Adelaide, Adelaide, SA Australia; 2 School of Biomedicine, The University of Adelaide, Helen Mayo North, Level 2, Room N237Frome Road, Adelaide, SA 5005 Australia; 3 Faculty of Humanities, Arts, Social Sciences and Education, University of New England, Armidale, NSW Australia

**Keywords:** Ned Kelly, Tattoos, Suicide, Homicide, Manner of death

## Abstract

Ned Kelly, an iconic figure in contemporary Australian mythology, was a bushranger (outlaw) who was executed in 1880 for the murder of a serving police officer, Constable Thomas Lonigan. Kelly is often commemorated by tattoos which depict his armour and helmet or his alleged last words of “Such is life”. A study was undertaken from January 1, 2011, to December 31, 2020, at Forensic Science SA, Adelaide, South Australia, of all cases with such tattoos. De-identified case details included the year of death, age, sex and cause and manner of death. There were 38 cases consisting of 10 natural deaths (26.3%) and 28 unnatural (73.7%). The latter included 15 cases of suicide (39.5%), 9 accidents (23.7%) and 4 homicides (10.5%). Of the 19 suicides and homicides, there were 19 males and no females (age range 24–57 years; average 44 years). The number of suicides in the general South Australian forensic autopsy population in 2020 was 216/1492 (14.5%) which was significantly lower than in the study population in which 39.5% of cases were suicides (2.7 times higher; *p* < 0.001). A similar trend occurred for homicides which accounted for 17/1492 in the general forensic autopsy population (1.1%), significantly lower than in the study population which had 10.5% homicides (approximately 9.5 times higher; *p* < 0.001). Thus, in the select population referred for medicolegal autopsy, there appears no doubt that Ned Kelly tattoos are associated with suicides and homicides. While this is not a population-based study, it may provide useful information for forensic practitioners dealing with such cases.

Australian history “does not read like history but like the most beautiful lies”.

Mark Twain (Samuel Clemens 1835-1910) [1]

## Introduction

**Fig. 1:**
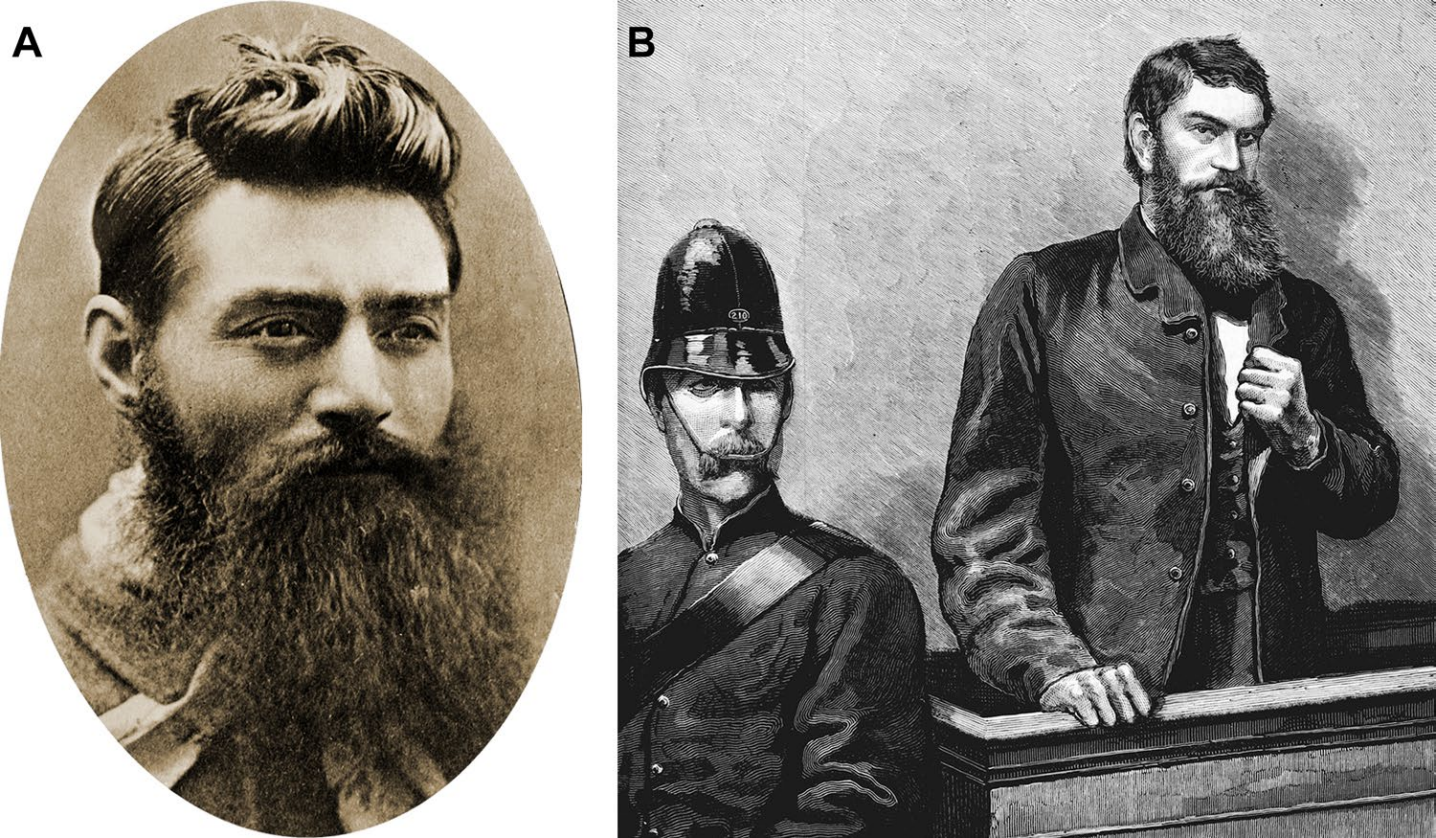
A photograph of Ned Kelly taken in 1880 **A** (National Archives of Australia, Public Domain) and a woodcut of his trial in Melbourne **B** (State Library of Victoria, Public Domain)

In early colonial Australia, the term “bushranger” referred to convicts who had escaped from custody to survive by foraging in the unsettled areas or “bush” [2]. Later, it was used as a term to describe outlaws who also took refuge in the bush, often in gangs, who robbed banks and farmhouses and who stole cattle and horses from sharecroppers. Ned Kelly (Fig. 1), one of the better-known bushrangers, was executed on 11th November 1880 for the murder of Constable Lonigan, although he and his gang also killed two other police officers, Sergeant Kennedy and Constable Scanlan, in a carefully orchestrated ambush at Stringybark Creek [3]. Kelly was later captured during a siege at Glenrowan where he had held up to 62 hostages in preparation for killing a number of police officers and Aboriginal troopers who had been despatched to the area by train [4]. Had he succeeded, it would have been the largest single massacre of police in Australia to date.

Over the years, two opposing views of Kelly have developed. Many maintain that he was a callous killer. Modern investigations and analyses have added some credence to these views. For example, it has been argued that Kelly did not fire as many rounds at Stringybark Creek, as scoring of his bullets (cutting an “x” into the soft lead) would have resulted in bullets fragmenting in the air, and thus more wounds would have occurred with fewer projectiles. This phenomenon was confirmed at a police firing range in the “Lawless – The Real Bushrangers” documentary series where a replica of Kelly’s gun was tested [5]. However, this does not address the question as to why Kelly scored his bullets in the first place. Such altered bullets are called “expanding bullets” and are known to fragment on hitting a target thus causing more tissue and organ damage. Their use was outlawed by the Hague Convention of 1899 [6].

A recent analysis of Kelly’s behaviour has also revealed disturbing trends with an assessment by a psychiatrist suggesting that Kelly was psychopathic and guilty of “pathological lying, callous lack of empathy for others and a parasitic lifestyle” [7]. Not only were his actions and police record reviewed but so were his comments. In the Jerilderie letter, for example, he said in reference to the police, “I would have scattered their blood and brains like rain. I would manure the Eleven Mile with their bloated carcasses”. He also vented his spleen against those who helped the police: “Without medicine I shall be compelled to make an example of some of them …….. pegged on an ant bed with their bellies opened, their fat taken out, rendered and poured down their throat boiling hot” [8]. In light of these sentiments, it is perhaps surprising that people continue to consider him a folk hero.

An alternative view of Ned Kelly as a champion of working-class oppression emerged in the early twentieth century. The first pro-Kelly book, *The Inner History of the Kelly Gang*, was published in Melbourne in 1929. The author, James Kenneally, was a journalist, trade unionist and founding member of the Country Labour Party. Kenneally’s popularisation of the Kelly story coincided with George Arnold Wood’s re-interpretation of the social origins of Australia’s convict migrants and the heightened social tensions which accompanied the Great Depression [9]. McQuilton was undoubtedly right when he argued that it was the economic hardship of the early thirties that gave the Kelly story “a new lease of life and a new significance” [10]. Ward’s attempt in the early 1950s to trace and explain Australian’s perceptions of their history further solidified Kelly’s identification as the “righter of wrongs” perpetuated by the authorities against the oppressed [11], an image that was reinforced through the painter Sidney Nolan’s many enigmatic works depicting Kelly in stylised armour.

A lasting consequence of this has been the casting of Kelly as a folk hero, a type of Robin Hood remembered by expressions in Australian parlance such as “as game [sic “bold”] as Ned Kelly” and regarded by some as “a key element of Australian identity” [2, 3]. Major sporting teams have had his image as part of their logos [1], and Ned Kelly figures borrowed from Nolan’s works featured prominently in the opening ceremony of the 2000 Sydney Olympic Games [12]. A stamp was issued by the Australian Commonwealth Government to commemorate the centenary of his death [13]. He has been referred to as “the prince of larrikins”, the latter term being used in Australia for “a mischievous young person, an uncultivated, rowdy but good hearted person” or even “a juvenile centaur” [14, 15]. These are all interesting, complimentary and yet puzzling memorials to a self-confessed and convicted thief and police killer [14].

In a previous retrospective study, the association of tattoos depicting Ned Kelly with a particular manner of death was examined, which showed that in a medicolegal environment, there was a significantly higher rate of unnatural deaths due to trauma in decedents who had these tattoos [16]. As this study was criticised because of its retrospective nature, the following prospective study was undertaken to clarify this issue.

## Materials and methods

A 10-year prospective study was commenced on 1st January 2011 and terminated on 31 December 2020 at the Forensic Science SA mortuary in Adelaide, South Australia. All cases with a tattoo depicting either Ned Kelly, his helmet or his alleged final words on the gallows of “Such is life” were registered in a de-identified manner, listing only the year of death, the age, sex and cause and manner of death. Statistical analyses were performed using standard Chi^2^ testing.

## Results

**Fig. 2:**
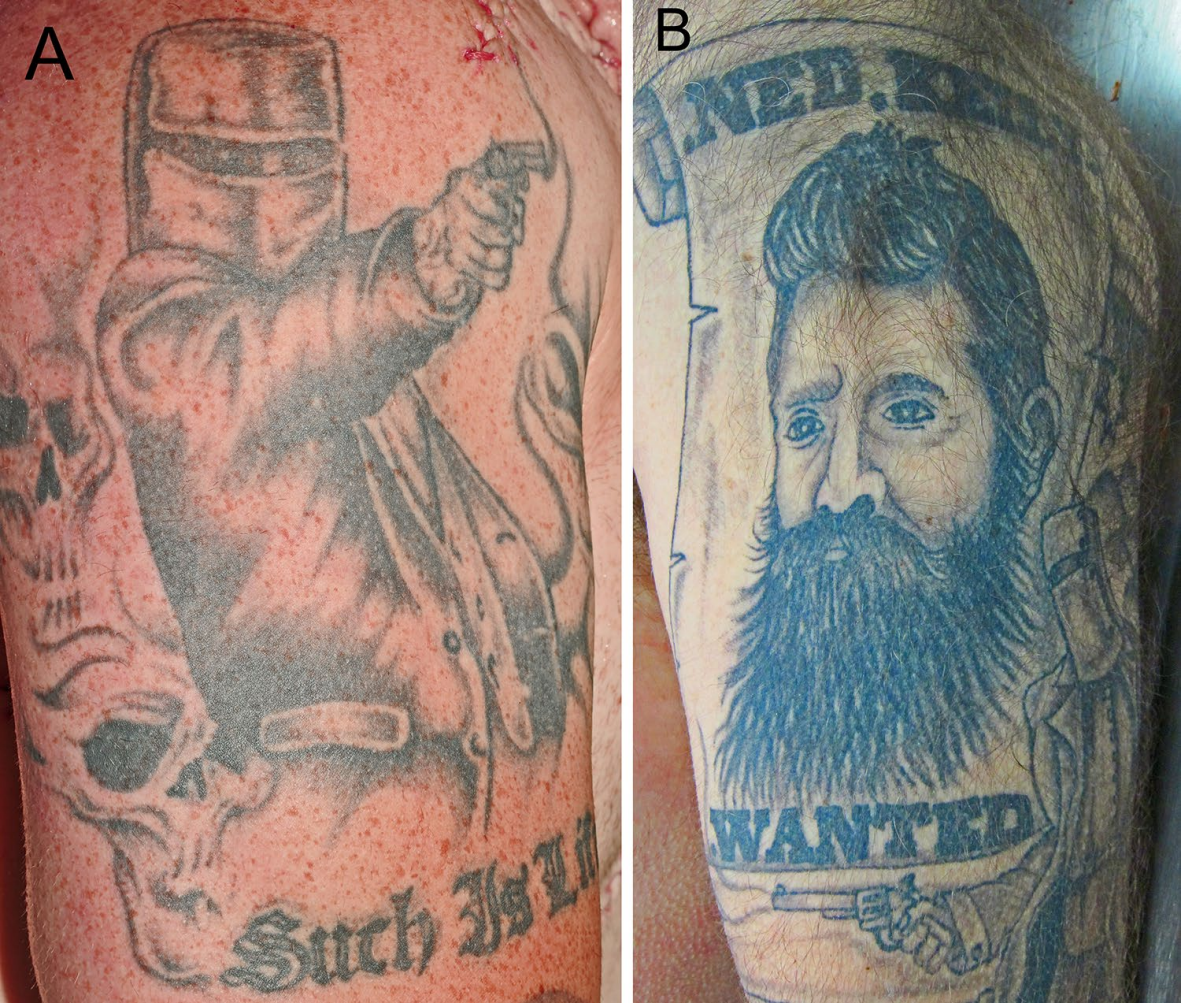
A quite sophisticated fine line tattoo depicting Kelly with his alleged last words (**A**) and a fine line portrait (**B**)

**Fig. 3:**
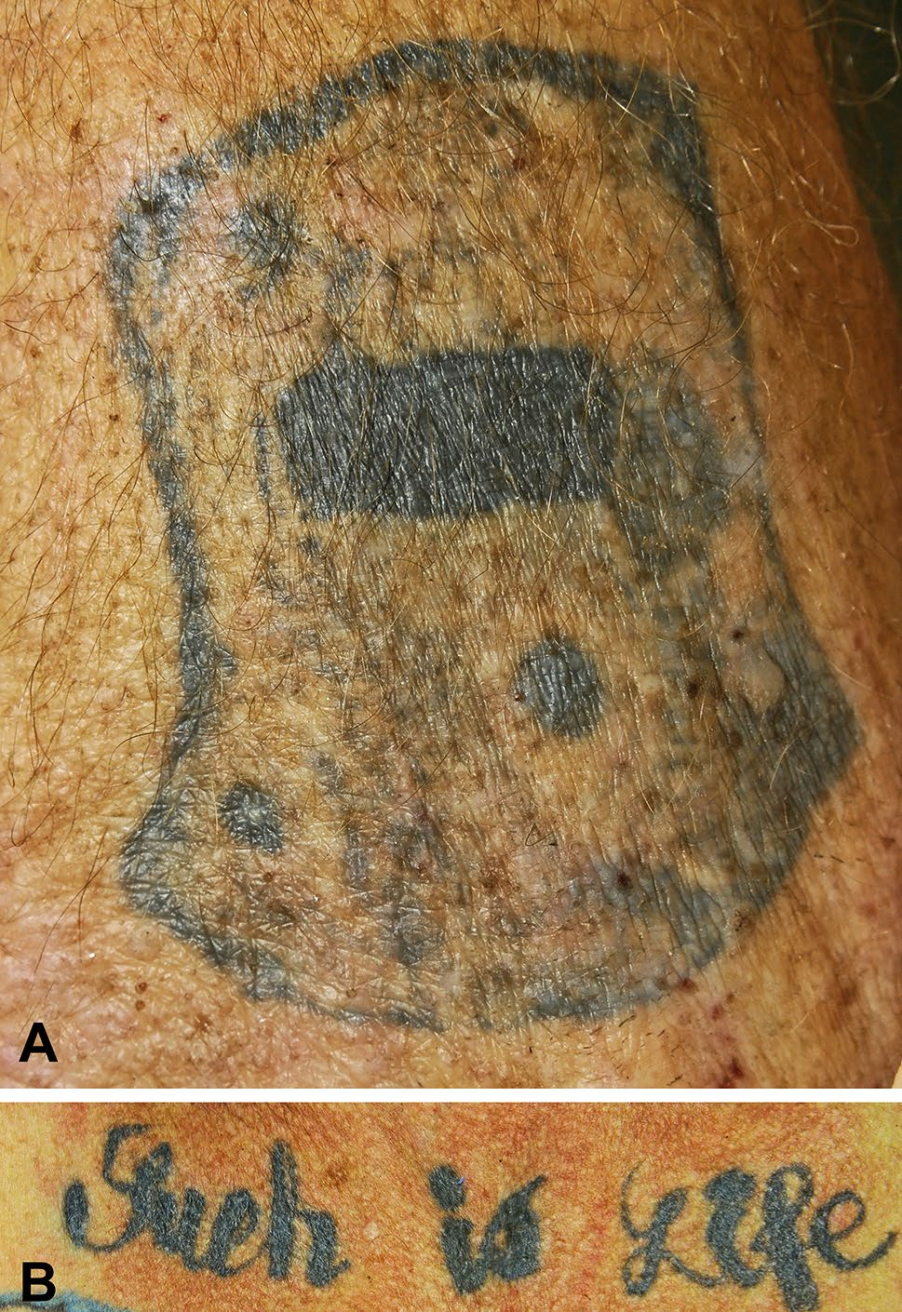
A primitive outline of Kelly’s helmet that he used at the Glenrowan siege (**A**), contrasting with tattoos with text and no images (**B**)

**Fig. 4:**
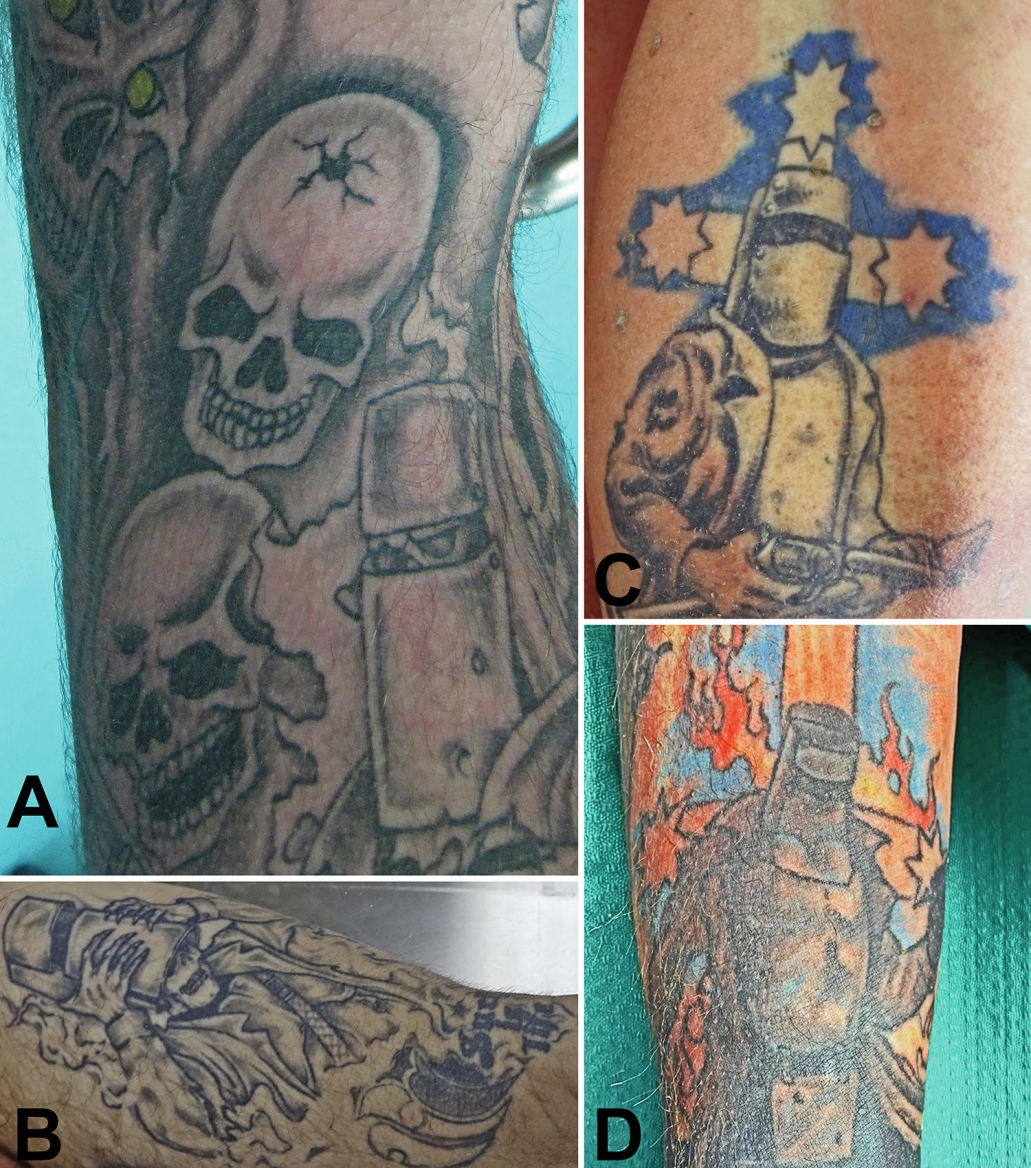
A tattoo of Kelly with skulls, one of which has a bullet hole in the cranium (**A**), one with his skeletonized head being revealed as his helmet is being removed (**B**) and another with a backdrop of the Eureka flag which is now used for racist purposes (**C**). In the final tattoo, the burning cross of the Eureka flag has been carefully positioned to suggest the crucifixion of a martyr or the flaming cross of a crusader (**D**)

**Fig. 5:**
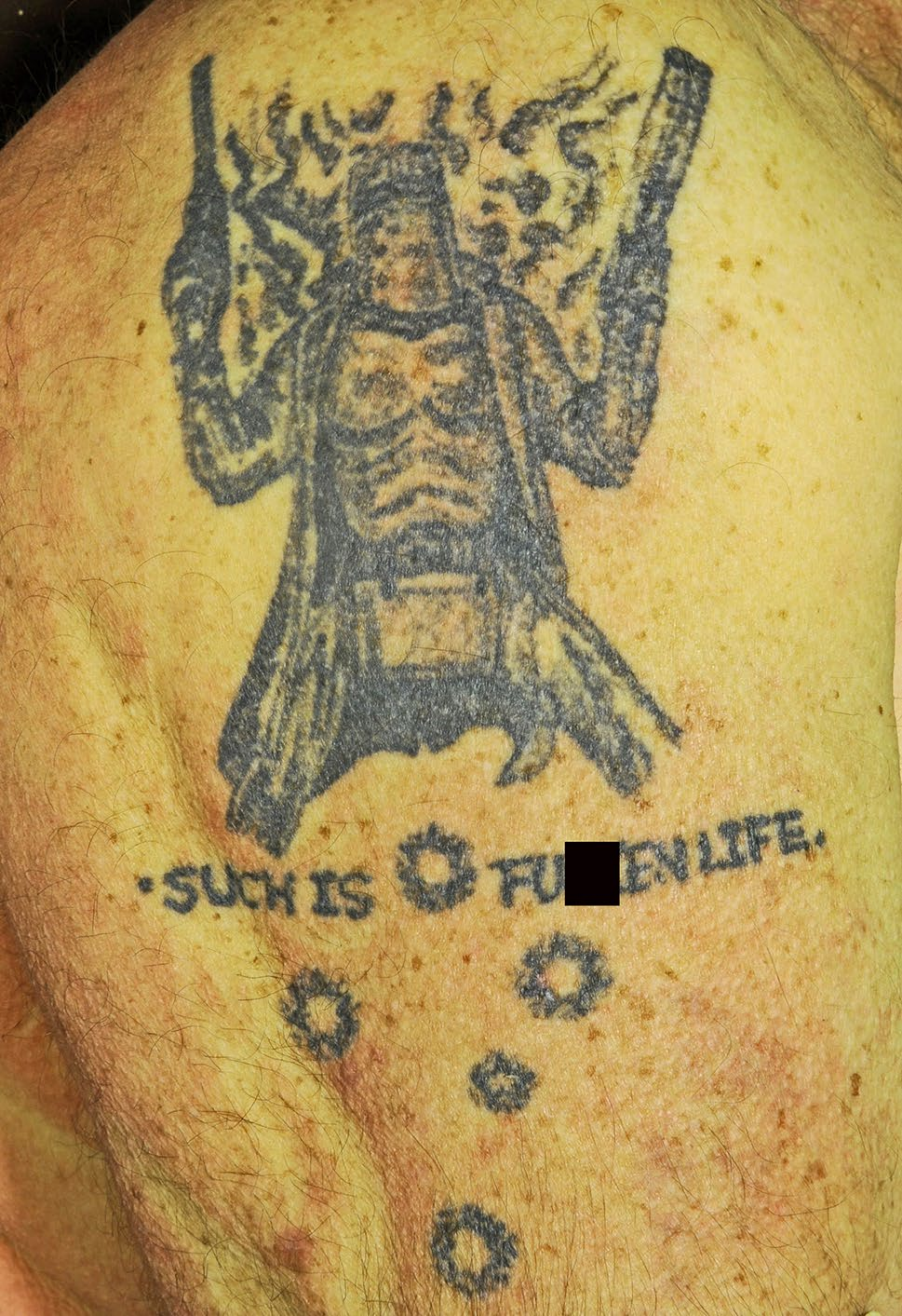
Augmentation of “Such is life” with an expletive (partly covered)

Tattoos varied from quite sophisticated standing figures brandishing weapons (Fig. 2) to primitive helmets or simply text (Fig. 3), with or without symbols of death such as skulls or racist symbols (Fig. 4). The words “Such is life” were sometimes augmented with expletives (Fig. 5).

There were 38 cases in the 10 years of the study with 10 cases of natural deaths (26.3%) and 28 unnatural (73.7%). The latter consisted of 15 cases of suicide (39.5%), 9 accidents (23.7%) and 4 homicides (10.5%). Of the 19 suicides and homicides, there were 19 males and no females, with ages from 24 to 57 years (average 44 years).

The number of suicides in the general forensic autopsy population of South Australia in 2020 was 216/1492 (14.5%) which was significantly lower than in the study population in which 39.5% of cases were suicides (2.7 times higher; *p* < 0.001). A similar trend occurred for homicides which accounted for 17/1492 in the general forensic autopsy population (1.1%), significantly lower than in the study population which had 10.5% homicides (approximately 9.5 times higher; *p* < 0.001).

## Discussion

Ned Kelly is most often depicted wearing a metal helmet and body armour (Fig. 4C) that he had constructed for protection during the planned massacre of police at Glenrowan [4]. Unfortunately for him, it did not cover his legs resulting in his subsequent wounding and capture. Subsequently, he has been “widely revered” and mythologised as an underdog who supported his friends and family against police oppression and social injustice. “Lionised as a bushranging hero” fighting against the tyrannical British^4^, he is often thought of as symbolizing a “romantic and rebellious aspect of Australian identity” [13, 17].

This may explain his depiction in contemporary tattoos. Tattoos are designs and patterns that are left in the skin after puncturing to allow for the introduction of pigmented dyes. The significance of tattoos has varied considerably amongst different societies and over time. Within the British Empire, compulsory tattooing was used to mark army deserters and Indian convicts. Rates of tattooing were particularly high amongst late eighteenth- and nineteenth-century sailors and soldiers as well as in prisoners and convicts transported to Australia [18, 19]. In Western culture, tattoos were commonly associated with membership of criminal gangs [20, 21]. In more recent years, the practice has gained wider social acceptance, and tattoos are now popular at all levels of society involving for example an estimated 10–16% of teenagers [22, 23].

This change in demographic profile means that there are now no differences in the manner of death between those who are tattooed and those who are not [24]. Similarly, the number of tattoos is also not predictive of the manner of death [25]. This does not, however, apply to antisocial tattoos such as those with expletive words or symbols such as swastikas, the latter in Western societies being associated with white supremacist sentiments. In both situations, there is an increase in violent and unnatural deaths in individuals who present for autopsy in a forensic context [26, 27]. Given the non-random nature of this medicolegal population, it is important to stress that this does not necessarily translate to the wider community.

Although tattoos depicting Ned Kelly often include the words “Such is life”, it appears unlikely that he actually uttered this phrase [28]. Other designs including his helmet or armour are often embellished with guns and skulls (Figs. 2, 4 and 5). The initial study into the possible relationship between Ned Kelly tattoos and the manner of death in cases undergoing forensic autopsies found a significant increase in the rates of both suicide and homicide in those with Kelly tattoos (2.7 and 7.7 times, respectively) [16]. The current study has shown similar findings, with 39.5% of cases being suicides compared to 14.5% in the general autopsy population (also 2.7 times higher; *p* < 0.001) and 10.5% homicides compared to 1.1% in the general autopsy population (approximately 9.5 times higher; *p* < 0.001). Given that this finding is undoubtedly influenced by factors such as socioeconomic status, mental health issues, criminal history and substance abuse, it would be useful for future studies to examine these specific factors in the context of such tattooing.

Thus, there appears no doubt that in a forensic context in Australia, Ned Kelly tattoos are markers for unnatural deaths such as suicides and homicides. It may be that the anti-authoritarian aspect of these tattoos puts them into the category of antisocial tattoos along with those having expletive words and phrases, or swastikas [26, 27]. Again, it must be reiterated that this is not a population-based study, and so the conclusions apply solely to the narrow population of individuals undergoing medicolegal autopsies. It is, however, a useful finding for forensic practitioners who may be required to evaluate and analyse such cases.
